# Nonexposure Accurate Location *K*-Anonymity Algorithm in LBS

**DOI:** 10.1155/2014/619357

**Published:** 2014-01-29

**Authors:** Jinying Jia, Fengli Zhang

**Affiliations:** School of Computer Science & Engineering, University of Electronic Science and Technology of China, Chengdu 611731, China

## Abstract

This paper tackles location privacy protection in current location-based services (LBS) where mobile users have to report their exact location information to an LBS provider in order to obtain their desired services. Location cloaking has been proposed and well studied to protect user privacy. It blurs the user's accurate coordinate and replaces it with a well-shaped cloaked region. However, to obtain such an anonymous spatial region (ASR), nearly all existent cloaking algorithms require knowing the accurate locations of all users. Therefore, location cloaking without exposing the user's accurate location to any party is urgently needed. In this paper, we present such two nonexposure accurate location cloaking algorithms. They are designed for *K*-anonymity, and cloaking is performed based on the identifications (IDs) of the grid areas which were reported by all the users, instead of directly on their accurate coordinates. Experimental results show that our algorithms are more secure than the existent cloaking algorithms, need not have all the users reporting their locations all the time, and can generate smaller ASR.

## 1. Introduction

In recent years, the consumer electronics markets witnessed a booming sale of smart mobile devices. These devices, typically smart phones and Personal Digital Assistants (PDA), are equipped with powerful CPU, large ROM and RAM, and positioning technology (e.g., GPS and AGPS). The omnipresence of these devices opens up new applications for the mobile users [1–3]. In particular, with the combination of GPS and wireless internet, mobile users can enjoy LBS, which provide dynamic content according to where the user is located. Typical LBS applications include the nearest point of interest (POI) query, location-aware advertisement, and road navigation. In order to enjoy such services, mobile users must explicitly expose their accurate locations to the server. For example, if the users ask for the nearest hospital, they must provide the LBS server with their accurate position in terms of GPS coordinates. In this sense, the privacy of the user's location is compromised in exchange for services. To solve this problem, an intuitive method is to cache the entire datasets of POIs on the mobile device, which can then resolve location-based queries locally. However, the resources of the mobile device are limited. This method can neither scale to large POIs datasets nor deal with data updates. Therefore, a more sophisticated strategy called location anonymity has been proposed and studied [4–7]. The purpose is to allow the mobile user to request services without revealing the accurate location. Among various approaches proposed along this line, location cloaking is predominant [4–6]. It blurs the user's accurate location and replaces it with a well-shaped cloaked region (usually a circle or a rectangle), according to some anonymity metric such as *k*-anonymity (the cloaked region must contain at least *k* users) or granularity (the size of the cloaked region must exceed a threshold). In effect, location cloaking achieves privacy protection at the cost of degrading service. The larger the cloaked region is, the more privacy is preserved, but the less precise the demand is. Therefore, most existent location cloaking research focuses on minimizing the size of the cloaked region while still satisfying the anonymity metric. To this end, a number of location cloaking algorithms have been proposed for different anonymity metrics [4, 5, 8]. However, to get the ASR and optimize its size, all the existent algorithms require the accurate locations (i.e., the coordinates) of all users. As the accurate locations are exactly what the users want to hide, all the existent work essentially imposes an assumption that all parties involved in the cloaking process must be trusted. Typical parties include the “anonymizer” that sits in between the user and the LBS server [5, 8]. However, in practice, any of these parties could be malicious and the exposure of the accurate location information to any party might reveal users' identity or other sensitive information. In this sense, existent algorithms have limited applications and location cloaking without exposing the accurate user location to any party is urgently needed.

Recently, a great deal of interest has been focused in the study of location privacy protection [4–14] and many of the references therein. But they exposure the users' accurate location to the third party. In this paper, we present such two nonexposure accurate location cloaking algorithms. They are designed for *k*-anonymity, and cloaking is performed based on the IDs of the gridareas which were reported by all the users.

To summarize, our contributions in this paper are as follows.

(i) In order to get such a cloaked region without exposing the accurate user locations, we propose two algorithms. One is Optimal cloaking algorithm and the other is random cloaking algorithm Both need not have all the users reporting the information of their positions all the time; they just report the information of their positions to the centralized anonymizer when they move to a new grid-area. To the best of our knowledge, this is the first study that explores the problem of location cloaking with the IDs of the grid-areas (not the users' accurate coordinates) which were reported by all the users, and the users can distinguish the grid-area which they are being in by themselves (not the centralized anonymizer).
(ii) Our algorithms can generate multiple ASRs for one query instead of a single ASR directly. We propose a measure model to measure the quality of service (QoS) of the grid-area. The optimal cloaking algorithm uses it to generate ASR with grid-area, which has the highest QoS. So the total square measure of the multiple ASRs is smaller than the single ASR which was generated by the existent cloaking algorithms.

The rest of the paper proceeds as follows.  Section 2 reviews existent work on location anonymity and privacy-aware LBS.  Section 3 presents the system architecture.  Section 4 presents the measure model for QoS and 2 generated ASR algorithms for the centralized environment. The experimental results are shown in Section 5 followed by the conclusion and future work in Section 6.

## 2. Related Work

Location anonymity has attracted intensive research as a solution to protect user privacy in mobile computing, especially for LBS. The purpose is to allow the mobile users to request services without disclosing their position. Among various anonymous techniques, location cloaking is the predominant. It sends to the server a cloaked region (usually a circle or a rectangle) that contains the genuine user position and is large enough to satisfy some privacy metric. The two most widely adopted metrics are *k*-anonymity—this region must contain at least *k* users so that the genuine requesting user is indistinguishable from at least *k* − 1 other users who have the same cloaked region—and granularity—the area of this region must exceed a threshold. Interval Cloak [4] is one of the first cloaking techniques. The anonymizer indexes the users with a quadtree. To form an ASR for the querying user, Interval Cloak descends the quad-tree up to the topmost node that contains at least *k* users (including querying user). The extent of this node is returned as the ASR. Casper Cloak [5] is similar to Interval Cloak, with two major differences. First, Casper Cloak identifies and accesses the leaf level of the quad-tree directly through the use of a hash table. Second, instead of immediately back tracking to the parent quadrant, it checks the two neighboring quadrants to see if their combination with the user quadrant contains *k* (or more) users. Gedik and Liu considered a personalized *k*-anonymity model and proposed Clique-Cloak, which constructs a clique graph to combine clients that can share the same cloaked region [9]. They addressed the issue when a client continuously requested location cloaking and developed an optimal cloaking technique to resist tracing analysis attacks [6].

It is noteworthy that all these cloaking approaches (i.e., Interval Cloak, Casper Cloak, and Clique-Cloak) require the user to expose the accurate coordinates to the trusted centralized anonymizer. Hu and Xu proposed a nonexposure cloaking algorithm to generate the ASR. The algorithm is performed based on the proximity information among mobile users, instead of directly on their coordinates [10]. The proximity information which was used by the algorithm is the WiFi received signal strength (RSS) of neighboring peers or the time difference of arrival (TDOA) of beacon signals from its peers (the shorter the closer). To the best of our knowledge, the WiFi RSS which the android smart phones received comes from the WiFi access points (AP). Usually the android mobile telephones do not work in AP mode. The TDOA can be only measured by the base stations. And the TDOA is just related to the mobile phone and the base station. So the mobile phone cannot use TDOA to determine the proximity information between its peers. So their algorithm cannot be used for the smart phones which adopted the android OS. Wu et al. proposed a Guess-Answer cloaking algorithm to generate ASR with the interaction between user and server [11]. The server guesses an ASR and gives it to the users. The users tell their relative directions of the guess ASRs to the server. The server guesses new ASRs with the answers and gives them to the users. The users answer it. This work continues until the users are in the guessed ASRs. Their algorithm can work without the third centralized anonymizer, but it does not belong to the *k*-anonymity, and the user's location can be gotten through the IP of the user.

On the server side, to support location cloaking, spatial query processing on cloaked regions has also been studied. The Casper framework proposed by Mokbel et al. consists of both an anonymizer and a query processor. The processor evaluates spatial queries over the cloaked regions and returns a superset of the results to the client for further filtering [5]. Cheng et al. proposed a similar framework based on location uncertainty [12], where the returned results are probabilistic results.

Besides location cloaking, other anonymous techniques have also been proposed. Pseudonym decouples the mapping between the user identity and the location so that the server only receives the location without the user identity. However, such a technique is limited to those location-based services that do not require the user's identity. In particular, the lack of user identity makes the billing of these services impossible. Dummy generates fake user locations (called dummies) and mixes them together with the genuine user location into the request [13]. However, by monitoring long-term movement patterns of the user, the server may distinguish the authentic location from dummies. You et al. enhanced this technique by generating consistent movement patterns for dummies in a long run [14]. More recently, Man et al. proposed SpaceTwist [7], where the user repeatedly issues kNN queries from dummies, which they called anchors, until the kNN result for the genuine location is guaranteed. Ghinita et al. proposed a similar framework that is based on private information retrieval (PIR) [15]. The framework partitions the space into grid cells and then the user requests the content within the cell where they are located. Thanks to PIR, the user can encrypt which cell is requested while receiving the correct content. By setting proper content for each cell, this framework can support approximate and exact NN queries. Furthermore, the framework is shown to guard against correlation attacks, but this framework does not belong to the *k*-anonymity.

## 3. System Architecture

In this section, we give the space cutting in our system, privacy threat model, and the system architecture.

In our algorithms, the space provided by the LBS system is divided into small rectangular areas. Such a rectangular area can be called grid-area. And all their width, and heights are the same. Every grid-area is marked by its ID. The ID is made up of *X*_id_ and *Y*_id_, so a grid-area can be marked as ID(*X*, *Y*). All the users' locations are marked by their coordinates *U*(*x*, *y*). The width of the area is Δ*x* which is given by the system. The height of the area is Δ*y* which is given by the system too. If the Δ*x* and Δ*y* are too small, it reduces the degree of anonymity. If the Δ*x* and Δ*y* are too big, it increases the network load. Usually, the Δ*x* and the Δ*y* are set by the most users' minimum requirement of ASR. The origin of the entire space is (*x*_0_, *y*_0_). When we have the *U*(*x*, *y*), we can calculate out the ID(*X*, *Y*) by (1). Consider

(1) $$\begin{array}{c} X_{\text{id}}=\frac{\left(x_{u}-x_{0}\right)}{\Delta_{x}}+1,\\ Y_{\text{id}}=\frac{\left(y_{u}-y_{0}\right)}{\Delta y}+1. \end{array}$$

**Definition 1**

The distance between the ID_*a*_(*X*_*a*_, *Y*_*a*_) and the ID_*b*_(*X*_*b*_, *Y*_*b*_) is a natural number which can be indicated by the larger one between |*X*_*a*_ − *X*_*b*_| and |*Y*_*a*_ − *Y*_*b*_| (|*a*| indicates the absolute value of *a*).

For example, the distance between the grid-area of ID(3,2) and the grid-area of ID(3,3) is the larger one between |3 − 3| and |2 − 3|; the result is 1. We do not use the Euclidean distance, because our distance of Definition 1 represents the number of layers, we can use it to expand the search layer by layer conveniently.

**Figure 1:**
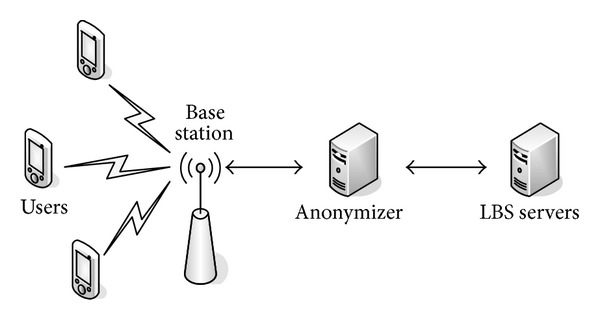
System architecture.

Figure 1 depicts the system architecture that consists of three entities: mobile users, anonymizer, and LBS server. We will first discuss our privacy threat model and privacy settings in user privacy profiles and then describe each entity in our system.

### 3.1. Privacy Threat Model

We assume that the centralized anonymizer is trusted, but not 100%. It does not leak the position information of the users, but it may be attacked by the adversary, and the adversary may capture the anonymizer, although the probability is very low. However, we do not have any assumption about the trustworthiness of the LBS providers (LBS servers).

### 3.2. User Privacy Profiles

All the users specify their privacy requirements in a privacy profile in a form of *K*, *A*_min⁡_, where *K* indicates the required anonymity level and *A*_min⁡_ indicates the required minimum area of their cloaked areas. In other words, the user wants to find an ASR that includes at least *K* users and has an area of at least *A*_min⁡_. It is important to note that the query user can change their privacy profile at the start time of any query to guarantee that her specified privacy settings achieve her desired privacy protection in different situations.

### 3.3. Mobile Users

All the mobile users are equipped with a wireless network interface card for communicating with the anonymizer, for example, GPRS, WCDMA, and CDMA2000. All the users are also equipped with a GPS or AGPS device to determine their location that is represented as a coordinate (*x*, *y*). All the users in the system can calculate the ID of the grid-area which they are being in by themselves (not the anonymizer) using (1). If they come into a new grid-area, they send their IDs of the new grid-areas and the IDs of the previous grid-areas which they have left to the anonymizer. When the users want to query something with *k*-anonymity, they send *K*, *A*_min⁡_, content of the query, and their grid-area IDs to the anonymizer. When the result of the query comes back from the anonymizer, they filter the useless POIs by their coordinates to get the last result.

### 3.4. Anonymizer

The anonymizer has a table or an array to record the numbers of users for each grid-area. When the users send their new ID, it updates the total of users in the previous grid-area and the new grid-area. It just records the total of users for each grid-area and does not record “which user is being in which grid-area.” So if the anonymizer is attacked by the adversary, it is very difficult for the adversary to know the accurate coordinates of the users. When the users want to query, the anonymizer generates the ASR for them, and then sends the query with the ASR to the LBS servers. The LBS server sends back the results of the query to the anonymizer. The anonymizer removes the useless information from the results by the query user's grid-area ID and then gives the useful information to the users.

### 3.5. LBS Servers

A privacy-aware query processor embedded inside the LBS servers has the ability to deal with location-based queries with multiple cloaked areas for one query. Since the query processor does not know the exact user location and which subASR the user is being in, it can only compute a candidate set (CS) of points of interest (POIs) for all the subASRs in the ASR which includes the exact answer for the user.

## 4. Generated ASR Algorithms

In this section, we give the main idea of the query algorithm at the LBS server, the measure model for QoS, two definitions, and two generated ASR algorithms.

### 4.1. Query Algorithm at LBS Server

**Figure 2:**
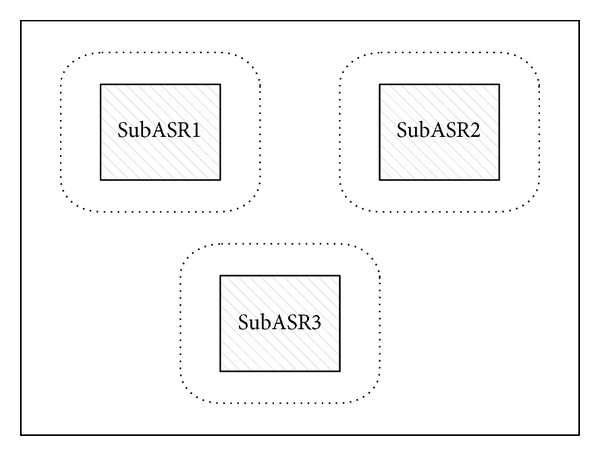
Principle of query algorithm at the LBS server.

Figure 2 depicts the principle of the query algorithm at the LBS server. The ASR of the query is made up of three isolated subASRs which are painted by the oblique lines. The LBS server does not know which subASR the user is being in, so it has to extend all the subASRs with a distance *R* to get the area for searching the POIs. The detail of the query algorithm at the LBS server oversteps the scope of this paper.

### 4.2. Measure Models

In order to reduce the size of CS, the total of the extended subASRs must be the smallest. The total of the extended subASRs is decided by the amount of the grid-areas and the distances between the grid-areas, so the grid-areas which are near to the query user's grid-area and have more users are the best for making the ASR.

If the total of users in the cloaked set of grid-areas *S* is not less than *K* (the degree of the anonymity for the user) and the total square measure of *S* is smaller than the *A*_min⁡_ (the smallest square measure of ASR required by the query user), the distance is the greater decisive factor. So we choose the grid-area which has the smallest sum of distances to each grid-area in *S*. The measure of the QoS is

(2) $$\begin{array}{c} \text{QoS}\;\left(\text{grid-area}\right)=\frac{1}{\text{sum}\left(d\right)}. \end{array}$$

If the total of users in *S* is less than *K*, in order to decrease the extended subASRs, the amount of users for the grid-area is the greatest decisive factor. The measure of the QoS is

(3) QoS (grid-area)={2∗usersK+1sum(d),users<K-sum(users  in  S)3+1sum(d),users≥K-sum(users  in  S).

When users in the grid-area are less than the difference between *K* and the total users in *S*, it means that we have to choose more than one grid-area to add to *S*. So the users and the distance are both the decisive factors. As the amount is more important than distance, we set the coefficient of the “users/*K*” to 2. So the QoS is 2∗users/*K* + 1/sum(*d*).

When users in the grid-area are not less than the difference between *K* and the total users in *S*, it means that we need to choose only one grid-area to add to *S*. So the QoS is just decided by the sum of distances. As the “2∗users/*K* + 1/sum(*d*)” is not greater than 3, in order to let the QoS be greater than “2∗users/*K* + 1/sum(*d*),” we let it be 3 + 1/sum(*d*).

### 4.3. Optimal ASR Algorithm

**Definition 2**

A rectangle can be indicated by Rec〈(*x*_ul_, *y*_ul_), (*x*_dr_, *y*_dr_)〉, when the (*x*_ul_, *y*_ul_) is the coordinate of the upper-left vertex of the rectangle and the (*x*_dr_, *y*_dr_) is the coordinate of the lower-right vertex of the rectangle.

**Definition 3**

A convex polygon can be indicated by Pol〈(*x*_1_, *y*_1_), (*x*_2_, *y*_2_),…, (*x*_*n*_, *y*_*n*_)〉, when the “(*x*_1_, *y*_1_), (*x*_2_, *y*_2_),…, (*x*_*n*_, *y*_*n*_)” are the coordinates of the vertexes of the convex polygon, and they must be sorted by clockwise or counterclockwise.

*Algorithm.* Algorithm 1 depicts the pseudo code of our optimal generating ASR. The anonymizer has a table of database or a two-dimensional array to record the total of users for each area. At most times, the querying users' grid-areas have enough users and are not less than the minimum area of the users required, so usually the ASRs of the querying users are their grid-areas. If there are not enough users in their grid-areas, it looks for the adjacent grid-areas. If there are enough users in the adjacent grid-areas, it adds the grid-areas which have the highest QoS to the cloaked set of grid-areas *S* until there are enough users in *S*. If there are not enough users in the adjacent grid-areas, it expands the radius to search more grid-areas nearby until all the effective grid-areas are searched. At last, if there are not enough users for all the effective areas, it reduces the degree of anonymity and lets *S* be the querying users' grid-areas; this situation is rare. If the total square measure of *S* is less than the *A*_min⁡_, it adds the grid-areas which have the highest QoS to *S* until the total square measure of *S* is not less than *A*_min⁡_. If there are grid-areas which are adjacent, it combines the adjacent grid-areas to the single area which can be indicated by a rectangle or a convex polygon. In the end, if there are grid-areas which are indicated by ID(*X*, *Y*) in *S*, it replaces them with Rec〈(*x*_ul_, *y*_ul_), (*x*_dr_, *y*_dr_)〉, because the LBS servers cannot understand the IDs of grid-areas. The areas in *S* are the query user's ASR.

**Figure 3:**
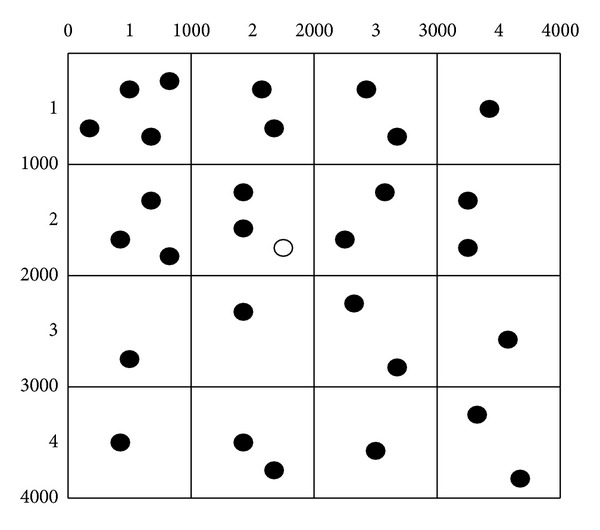
An example of the optimal algorithm.

Figure 3 depicts an example of our optimal algorithm. The width and the height of the grid-areas are both 1000. The hollow dot is the querying user. The solid dots are the other users. If the *k* = 3 and *A*_min⁡_ = Δ*x*∗Δ*y*, the querying user's grid-area has three users and is not less than *A*_min⁡_, so the ASR will be the querying user's grid-area. After replacing the ID(*X*, *Y*) of the grid-area with Rec〈(*x*_ul_, *y*_ul_), (*x*_dr_, *y*_dr_)〉, the result can be indicated by ASR = {Rec〈(1000,1000), (2000,2000)〉}. If *k* = 3 and *A*_min⁡_ = 2∗Δ*x*∗Δ*y*, then the querying user's grid-area is less than *A*_min⁡_, so it adds the grid-area which has the highest QoS to *S*. Since there are eight grid-areas having the same QoS, it selects the grid-areas randomly. If it selects the ID(2,3), then *S* = {ID(2,2), ID(2,3)}. As the ID(2,2) and the ID(2,3) are adjacent grid-areas, so they can be combined to a rectangle. The result can be indicated by ASR = {Rec〈(1000,1000), (2000,3000)〉}. Similarly, if *k* = 21 and *A*_min⁡_ = Δ*x*∗Δ*y*, our algorithm gets the last result which can be indicated by ASR = {Pol〈(0,0), (3000,0), (3000,1000), (4000,1000), (4000,2000), (3000,2000), (3000,3000), (0,3000)〉}.

Our Optimal algorithm generates the ASR using the grid-areas which have the highest QoS, so the ASR generated by our Optimal algorithm is smallest among all the existent cloaking algorithms [4, 5]; the experiments will verify this. So our Optimal algorithm is optimal.

### 4.4. Random ASR Algorithm

The optimal ASR algorithm can generate an ASR which has higher QoS, but if the adversaries knows the tactics of our generating ASR algorithm, they can know the query user's grid-area. It will decrease the security of the query user. For example, in line 5 of Algorithm 1, the distance *d* is initialized to 1. When it runs to line 7 of Algorithm 1, the value of *d* is 2. So the 24 grid-areas nearby the user's grid-area can be chosen. In fact, the distribution of users is not uniform. Usually, there are a lot of users in some small areas, such as the bus stations and cinemas. If the ASR is {ID(1,1), ID(1,3)} in Figure 3 and there is a bus station in ID(1,1), the adversary can know the user is in ID(1,3), because there may be a lot of users in ID(1,1) as there is a bus station. If the user is in ID(1,1), the ASR may be two grid-areas, which are adjacent as Algorithm 1. But the two grid-areas “ID(1,1), ID(1,3)” are not adjacent; that means “the user is in ID(1,1)” is not true. If the adversary can guess the subASR which the query user is being in, the other subASRs in the ASR will be useless.

*Algorithm.* Algorithm 2 depicts the pseudo code of our random generating ASR. In order to lead randomness into the algorithm, it creates “random” integer and sets its value randomly from 1 to 10. When it adds the grid-area to *S*, it judges whether “random” is greater than “rnd” (“rnd” is a threshold). If it is true, it adds the grid-area which has the highest QoS to *S*, or it adds the grid-area to *S* randomly. Usually, the threshold “rnd” is set to 2 or 3.

## 5. Experiment

In this section, we evaluate the performance of our proposed algorithms. The results of our algorithms are compared with Interval cloaking algorithm and Casper cloaking algorithm.

### 5.1. Experiment Settings

In all the experiments, we use the Network-based Generator of Moving Objects [16] to generate moving objects using a real road network. The actual query examples (*K* nearest neighbor query) are used for the experiments. The target objects are uniformly distributed in the space. The initial locations and moving trajectories of all the clients are constrained by the road network. Unless specified, our simulation is running over 5 K clients and 500 static query targets. All the clients can use their own GPS devices to get their coordinates. The speeds of all the users fall in the range of [0,40] km per hour. The probability of each user sending LBS requests follows an exponential distribution.

The space of our algorithm is divided into grid-areas. Both the width and height are 2 km. The anonymizer of our algorithm records the total users of each grid-area by a two-dimensional array or a table of the database. While the space of Interval cloaking algorithm and Casper cloaking algorithm is divided into multiple cells, the size of the cell is the same as our grid-area, and the anonymizer of the original algorithms maintains the cells using a quadtree structure.

### 5.2. Security Experiments and Theoretical Comparison

The purpose of our algorithms is to resolve the securities of the anonymizer. We use two computers to act as “the centralized anonymizer for Casper cloaking algorithm [5]” and “the centralized anonymizer for our cloaking algorithms”; we pretend to be the attackers to attack the two centralized anonymizers.

For the Casper cloaking algorithm [5], if we capture the anonymizer, we can know the accurate coordinates of all the users, because all the users report their accurate coordinates to the anonymizer, and the anonymizer records the accurate coordinates. Although the anonymizer may use the pseudonyms of the users, the information of accurate coordinates of all the users is very helpful for the adversary to guess the real users of the query and their accurate coordinate. For example, if Jack knows Jim has sent a query at his workplace and Jack has captured the anonymizer, the anonymizer will generate an ASR for Jim. If there is only one user at Jim's workplace, although there are *k* users in the ASR, Jack can guess the pseudonym of Jim easily. If Jim sends a query of the nearest help center of AIDS later, Jack will know that Jim may be suffering from AIDS. So do all the queries of Jim later.

For our algorithms, if we capture the anonymizer, we cannot know the accurate coordinates of all the users, because the anonymizer just records the total users for each grid-area, it does not record “which user is being in which grid-area.” It just records the grid-area ID of the query user temporarily. It erases this information after sending back the CS to the user. So we can only get the grid-area ID of the querying user and the total users of the querying user's grid-area. Usually the width and height of the grid-area are about 2 km, so it is very difficult to get the accurate coordinates of the querying user. The adversary can know nothing at all about the accurate coordinates of other users. What a pity! The anonymity level of the querying user should be reduced to the total users in the querying user's grid-area. However, it compares to the Casper cloaking algorithm [5]; our algorithms have improved the securities of the anonymizer.

### 5.3. Performances of Centralized Environment

**Figure 4:**
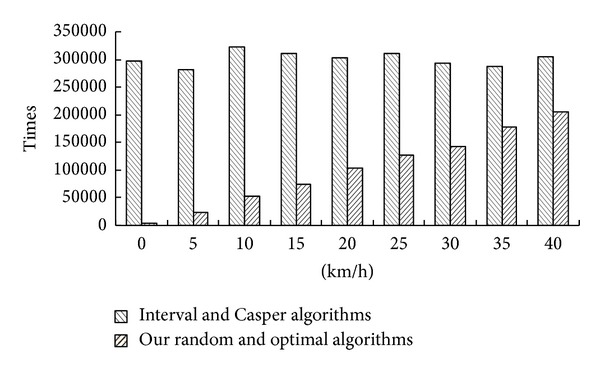
Report times in one hour.

The times of all the users' report to the anonymizer are an important factor for the performance of the centralized anonymizer. The more times reported, the poorer performance of the centralized anonymizer.  Figure 4 depicts the times of the 10 K users' report to the anonymizer during one hour at different velocities from 0 to 40 km/h for our Random algorithm, our optimal algorithm, the interval algorithm [4], and Casper algorithm [5] (the Casper algorithm uses the same strategy as the Interval algorithm for reporting the location, so we put them together). We can see the report times of our algorithms are smaller than the interval algorithm and Casper algorithm. It is very obvious when the velocity is low. The times and the velocity are the direct ratios in our algorithms, while the times in the interval algorithm and Casper algorithm are just constant.

Because the Interval [4] and Casper [5] cloaking algorithms cannot judge the cells by themselves, they must report at a specified frequency. While our two algorithms can judge the grid-areas by themselves, they just report to the centralized anonymizer when they come to a new grid-area. Reducing the report times is not only helping to improve the performance of the centralized anonymizer but also can save the resources of the wireless communication.

**Figure 5:**
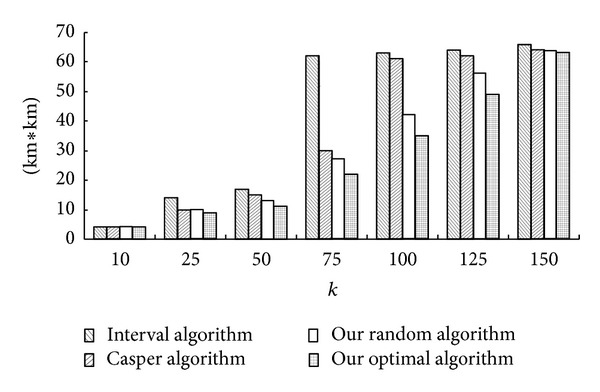
The square measure of ASR.

The square measure of ASR is an important factor for the performance of the LBS servers. The greater the ASR is, the poorer the performance of LBS servers, because LBS servers have to cope with more useless areas in the ASR, and the CS contains more useless POIs.  Figure 5 depicts the average square measure of ASR generated by the Interval Cloak algorithm, Casper Cloak algorithm, our Random algorithm and our Optimal algorithm when *k* is from 10 to 150, *A*_min⁡_ = 4 km^2^, and the threshold of randomness in our random algorithm is 2.

We can see clearly that the square measures of ASR generated by our two algorithms are not greater than the interval cloaking algorithm [4] and the Casper cloaking algorithm [5] from the bar graph, because when the value of *k* is small, the grid-area which the user is being in has enough users. The user's grid-area is the ASR. The grid-area is the same as the cell of the Interval cloaking algorithm [4] and the Casper cloaking algorithm [5], so the results are equal. When the value of *k* is a little greater, there are not enough users in the user's grid-area. The interval cloaking algorithm [4] has to find the father node for help. The Casper cloaking algorithm [5] can search its adjacent cells from its brotherly nodes. Our 2 algorithms can utilize any nearby grid-areas. So the results of the Casper cloaking algorithm [5] is larger; the results of the Interval cloaking algorithm [4] is largest; the results of our Random algorithm is smaller; the results of our Optimal algorithm is the smallest. The smaller the ASR is, the smaller the CS is. So our algorithms can save the resources of the communication between the centralized anonymizer and the LBS server.

## 6. Conclusion and Future Work

In general, we proposed two algorithms for nonexposure accurate location *k*-anonymity in LBS. The centralized anonymizer gave the origin of the entire space (*x*_0_, *y*_0_), the width of area Δ*x*, and the height of area Δ*y* to all the mobile users in the system. Mobile users judged their grid-areas IDs by themselves and reported their grid-areas IDs instead of their accurate coordinates to the anonymizer. So when the anonymizer was captured by the adversaries, the adversaries could not get the accurate coordinates of all the users (including the querying user) as easly as the existent algorithms. When the mobile users wanted to query, the anonymizer generated the ASR, which was composed of grid-areas and was expressed by the real coordinates instead of the grid-areas' IDs. The anonymizer sent the ASR instead of the query users' grid-areas IDs to the LBS server. When the query result came back to the anonymizer, the anonymizer filtered out the useless POIs by the query user's grid-areas ID and sent the result to the query users. When the query user received the result from the anonymizer, they filtered the useless POIs by their coordinates to get the last result.

It was a pity that our nonexposure accurate location *k*-anonymity algorithms could not resist the attack of continuous queries, could not satisfy the road network environment [17], and did not solve the single point of failure and scalability issues of the centralized anonymizer. How to improve our nonexposure accurate location anonymity algorithms to meet these needs is our work in the future.

## Figures and Tables

**Algorithm 1:**
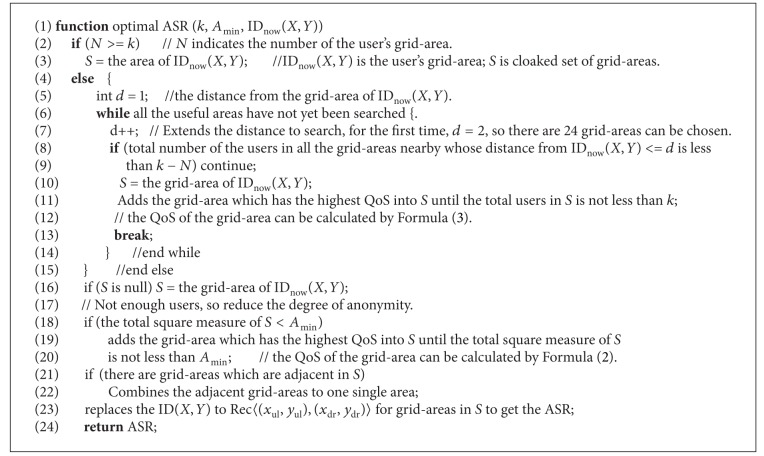
Optimal ASR.

**Algorithm 2:**
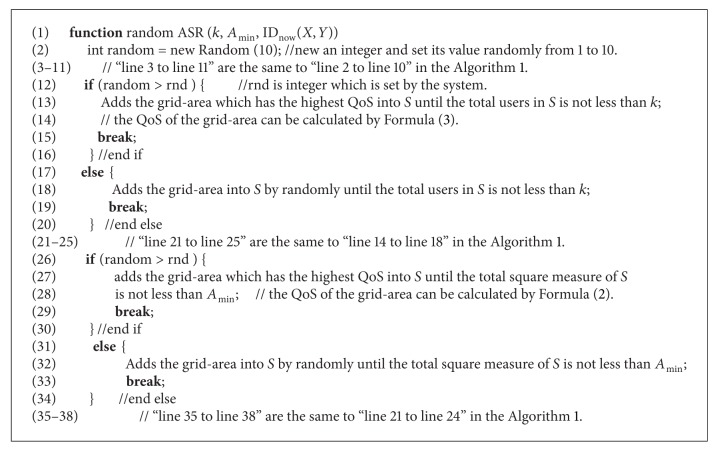
Random ASR.
